# Avoiding Traumatic Injury to the Tissues in the New Millennium

**DOI:** 10.5812/kowsar.22517464.3495

**Published:** 2012-01-15

**Authors:** Seyed Mehdi Jafari

**Affiliations:** 1 Department of Oral and Maxillofacial Surgery, Azad University Dental Branch, BouAli Medical Center, Tehran, IR Iran

**Keywords:** Wounds and Injuries, Tissues

*Dear Editor,*

While the world of surgery and medicine is racing towards endoscopic less invasive surgical techniques, we here are still quoting the 19th century adage “great surgeons make great incisions”. I teach oral and maxillofacial surgery at both undergraduate and postgraduate levels and can attest to the fact that minimally invasive procedures can maximize patient comfort by avoiding traumatic injury to the tissues. For example in my field, treatment modalities for plating of orbital and condylar fractures, antral explorations etc. have been done less traumatically using endoscopic surgery. In other fields, bronchoscopic procedures, laparoscopic removal of tissues, transurethral approaches to enlarged prostate etc. are being used indicative of a rapid shift towards less aggressive, noninvasive methods gaining acceptance. The role of diagnosis technology and computer-aided surgery is very relevant, and magniﬁcation technology with the assistance of computer technology, work together to provide more precise treatments offering patients a less traumatic experience.

The technology of computerized navigation has been developed in medicine for brain, ENT and orthopedic surgery. We have borrowed the concept of minimally invasive surgery (MIS) from other medical ﬁelds, as it has its greatest implications in the field of maxillofacial surgery. MIS involves smaller and more accurate incisions, using magniﬁcation systems and microsurgery instruments. MIS also requires fewer operations, which implies a very precise planning and a combination of surgical techniques aiming at several objectives simultaneously. In my field, ideal positioning of dental implants makes use of three-dimensional orientation technology to define accurately the mandibular canal or maxillary sinus, zygoma and pterygoid implant placement sites, roots of neighboring teeth as well as the optimum esthetic location to place the implant. Flapless insertion of dental implants can prevent complications arising from soft-tissue elevation such as infection, dehiscence and necrosis, and provides dental implant success rates equal to conventional techniques (1). In the anterior mandible, the flapless technique requires surgical guidance for optimal tilt of drilling to avoid injury to underlying anatomical structures during preparation of the implant socket. Computer-assisted surgery is known to enhance safety in dental implantology while being compatible with all aspects of implant surgery including flapless techniques (2). Flapless implant surgery has improved both the osseointegration of dental implants and the bone height around implants. Many investigators have emphasized that flapless implant surgery is a predictable procedure with high success rates if patients are appropriately selected and an appropriate width of bone is available for implant placement. The preservation of bone vascularization when no flaps are reflected may help optimize bone regeneration around implants.As it is now believed by some authors that flapless placement of implants cannot be successfully fulfilled in all the regions of the jaw bones, taking advantage of the new 3-D computerized navigation technologies, will certainly add to state-of-the art surgery.
